# Telocytes in normal and keratoconic human cornea: an immunohistochemical and transmission electron microscopy study

**DOI:** 10.1111/jcmm.13270

**Published:** 2017-07-17

**Authors:** Mirca Marini, Rita Mencucci, Irene Rosa, Eleonora Favuzza, Daniele Guasti, Lidia Ibba‐Manneschi, Mirko Manetti

**Affiliations:** ^1^ Department of Experimental and Clinical Medicine Section of Anatomy and Histology University of Florence Florence Italy; ^2^ Eye Clinic Department of Surgery and Translational Medicine University of Florence Florence Italy

**Keywords:** telocytes, stromal cells, human cornea, keratoconus, immunohistochemistry, transmission electron microscopy

## Abstract

Telocytes (TC) are typically defined as cells with telopodes by their ultrastructural features. Their presence was reported in the interstitium of various organs in vertebrates, including humans. However, no study has yet described the presence of TC in the human eye and in particular, within the stromal compartment of the cornea. To address this issue, samples of normal and pathologic (keratoconic) human corneas were tested by immunohistochemistry for CD34, platelet‐derived growth factor receptor α (PDGFRα) and c‐kit/CD117 or examined by transmission electron microscopy. We found that TC coexpressing CD34 and PDGFRα were distributed throughout the whole normal corneal stroma with different TC subtypes being distinguishable on the basis of the expression of the stemness marker c‐kit (*i.e*. c‐kit‐positive and c‐kit‐negative TC subpopulations). Transmission electron microscopy examination confirmed the existence of spindle‐shaped and bipolar TC typically displaying two long and thin moniliform telopodes establishing intercellular contacts formed by gap junctions. Keratoconic corneas were characterized by ultrastructural damages and patchy loss of TC with an almost complete depletion of the c‐kit‐positive TC subpopulation. We propose that TC may contribute to the maintenance of corneal stromal homoeostasis and that, in particular, the c‐kit‐positive TC subtype might have stemness capacity participating in corneal regeneration and repair processes. Further studies are needed to clarify the differential roles of corneal TC subtypes as well as the possible therapeutic applications of TC in degenerative corneal disorders such as keratoconus.

## Introduction

The cornea is an optically clear tissue that forms the front ocular surface and accounts for nearly two‐thirds of the eye refractive power 1. It consists of three cell layers: an outer epithelium, a middle avascular stromal layer formed by a collagen‐rich extracellular matrix (ECM) interspersed with interstitial cells and an inner layer of endothelial cells. The aforementioned layers are bordered by two membranes, namely the Bowman's membrane between the epithelial layer and the stromal layer, and the Descemet's membrane between the stromal layer and the endothelium 2, 3. Cornea must be transparent and maintain a smooth and stable curvature as it contributes to the major part of the focusing power of the eye. Corneal transparency is conferred by the highly organized ECM of the corneal stroma which represents more than 90% of the corneal thickness. In the corneal stroma, parallel bundles of collagen fibres are tightly packed in lamellae lying parallel to the corneal surface 2.

Corneal stromal cells, generally referred to as keratocytes, are interspersed between the collagen lamellae and are believed to play an important role in the preservation of corneal transparency and mechanical stability through the synthesis and/or maintenance of the ECM 4. Keratocytes have been described as cells displaying a dendritic‐like morphology characterized by a compact cell body with numerous cytoplasmic lamellipodia which establish intercellular junctions forming a three‐dimensional network 4, 5. As far as keratocyte immunophenotype is concerned, these cells are commonly identified by immunohistochemistry for the CD34 antigen 6, 7, 8. Moreover, these cells are known to undergo morphological and quantitative changes in corneal pathologies such as in keratoconus, a non‐inflammatory ectatic disorder characterized by a reduction in keratocyte density mainly due to increased apoptotic cell death 6, 7, 9.

A new type of interstitial cells, named telocytes (TC), has been recently described in the stromal compartment of many organs in vertebrates including humans 10, 11, 12, 13, 14, 15, 16, 17, 18, 19, 20, 21, 22. Moreover, this peculiar stromal cell type is being increasingly implicated in a wide range of pathologies with potential applications in regenerative medicine 23, 24, 25, 26, 27, 28, 29, 30, 31, 32, 33, 34, 35, 36. Telocyte possess unique ultrastructural features consisting of a small cell body and extremely long and thin prolongations, termed telopodes, displaying a moniliform aspect characterized by the alternation of thin segments (called podomers) and small dilated regions (called podoms) accommodating mitochondria, endoplasmic reticulum cisternae and caveolae 11, 12, 37. Telopodes are typically organized in a three‐dimensional network which comprises either homocellular junctions between TC or heterocellular communications between TC and other cell types 11, 12, 38, 39. Moreover, TC are believed to participate in intercellular signalling through the release of different types of extracellular vesicles, such as exosomes, ectosomes and multivesicular cargos 12, 40, 41, 42. Although TC do not possess a unique antigenic profile, a combination of CD34 and platelet‐derived growth factor receptor α (PDGFRα) is currently considered the most reliable marker for their immunohistochemical identification 12. Indeed, coexpression of CD34 and PDGFRα has been widely observed in TC from different human organs 12, 43, 44. It also appears that the TC immunophenotype may vary among systems and that different TC subtypes likely playing differential roles may coexist within the same organ 45, 46, 47, 48, 49. For instance, TC may express either CD34, PDGFRα or c‐kit/CD117 in some human organs, such as the heart, while they are CD34^+^/PDGFRα^+^ and c‐kit‐negative in others, such as the gastrointestinal tract [25, 43, 44, 45, 47].

To the best of our knowledge, to date, no study has described the possible presence of TC within the stromal compartment of the human cornea. Considering the striking similarities between some of the morphological and immunophenotypical features of TC and those described for the so‐called corneal keratocytes 1, 4, 5, 6, here we were prompted to investigate whether TC may represent a cellular component of the human corneal stroma. To this aim, we carried out a series of immunohistochemical and ultrastructural studies on human corneal specimens, including a comparison between normal and keratoconic corneas.

## Materials and methods

### Human corneal specimens

The study was carried out on six normal human corneas from Eye Bank and six human corneal buttons from patients with keratoconus without subepithelial scarring removed during penetrating keratoplasty, as described elsewhere 6. All the patients signed a written informed consent form, and the study was carried out in accordance with the Declaration of Helsinki and approved by the Institutional Review Board. Central corneal buttons of similar diameter were analysed. Immediately after removal, the corneal buttons were cut in small pieces which were processed for light, fluorescence and transmission electron microscopy.

### Immunoperoxidase‐based immunohistochemistry

Immunohistochemical studies were performed on deparaffinized and rehydrated corneal sections (5 μm thick). Corneal tissue sections were boiled for 10 min. in sodium citrate buffer (10 mM, pH 6.0) for antigen retrieval and treated with 3% hydrogen peroxide solution for 15 min. at room temperature to block endogenous peroxidase activity. Sections were then washed and incubated with Ultra V block (UltraVision Large Volume Detection System Anti‐Polyvalent, HRP, catalogue number TP‐125‐HL; Lab Vision, Fremont, CA, USA) for 10 min. at room temperature according to the manufacturer's protocol. After blocking non‐specific site binding, slides were incubated overnight at 4°C with mouse monoclonal anti‐human CD34 antibody (1:50 dilution; clone QBEnd‐10, catalogue number M7165; Dako, Glostrup, Denmark). The day after, tissue sections were washed three times in PBS and subsequently incubated with biotinylated secondary antibodies (UltraVision Large Volume Detection System Anti‐Polyvalent, HRP; Lab Vision) for 10 min. at room temperature. The slides were then washed three times in PBS and incubated with streptavidin peroxidase (UltraVision Large Volume Detection System Anti‐Polyvalent, HRP; Lab Vision) for 10 min. at room temperature. Immunoreactivity was developed using 3,3′‐diaminobenzidine tetrahydrochloride as chromogen (Sigma‐Aldrich, St. Louis, MO, USA). Corneal sections were finally counterstained with Mayer's haematoxylin (Bio‐Optica, Milan, Italy) and observed under a Leica DM4000 B microscope equipped with fully automated transmitted light axes (Leica Microsystems, Mannheim, Germany). Sections not exposed to primary antibodies or incubated with isotype‐matched and concentration‐matched non‐immune mouse IgG (Sigma‐Aldrich) were included as negative controls for antibody specificity. Light microscopy images were captured with a Leica DFC310 FX 1.4‐megapixel digital colour camera equipped with the Leica software application suite LAS V3.8 (Leica Microsystems).

### Immunofluorescence staining

Double immunofluorescence staining combining CD34 either with PDGFRα or c‐kit/CD117 was performed according to previously published studies 31, 32, 43. Paraffin‐embedded corneal tissue sections (5 μm thick) were deparaffinized, rehydrated and boiled for 10 min. in sodium citrate buffer (10 mM, pH 6.0). Sections were washed in PBS, incubated in 2 mg/ml glycine for 10 min. to quench autofluorescence caused by free aldehydes and then blocked for 1 hr at room temperature with 1% bovine serum albumin (BSA) in PBS. The sections were then incubated overnight at 4°C with the following primary antibodies diluted in PBS with 1% BSA: mouse monoclonal anti‐human CD34 (1:50 dilution; clone QBEnd‐10, catalogue number M7165; Dako), goat polyclonal anti‐human PDGFRα (1:100 dilution; catalogue number AF‐307‐NA; R&D Systems, Minneapolis, MN, USA) and rabbit polyclonal anti‐c‐kit/CD117 (1:200 dilution; catalogue number A4502; Dako). The day after, the slides were washed three times in PBS and incubated for 45 min. at room temperature in the dark with Alexa Fluor‐568‐conjugated donkey anti‐mouse IgG, Alexa Fluor‐488‐conjugated donkey anti‐goat IgG or Alexa Fluor‐488‐conjugated goat anti‐rabbit IgG (Invitrogen, San Diego, CA, USA) diluted 1:200 in PBS with 1% BSA, as secondary antibodies. Double immunofluorescence staining was performed by mixing mouse and rabbit or goat primary antibodies and subsequently mixing appropriate fluorochrome‐conjugated secondary antibodies. Irrelevant isotype‐matched and concentration‐matched mouse, rabbit and goat IgG (Sigma‐Aldrich) were used as negative controls. Cross‐reactivity of secondary antibodies was tested in control experiments in which primary antibodies were omitted. Nuclei were counterstained with 4′,6‐diamidino‐2‐phenylindole (DAPI; Chemicon International, Temecula, CA, USA). Corneal sections were then mounted with an antifade aqueous mounting medium (Biomeda Gel Mount; Electron Microscopy Sciences, Foster City, CA, USA) and examined with a Leica DM4000 B microscope equipped with fully automated fluorescence axes (Leica Microsystems). Fluorescence images were captured with a Leica DFC310 FX 1.4‐megapixel digital colour camera equipped with the Leica software application suite LAS V3.8 (Leica Microsystems).

### Quantitative analysis

Quantitative analysis of TC was performed on corneal tissue sections immunostained with the mouse monoclonal anti‐CD34 antibody and the rabbit polyclonal anti‐c‐kit/CD117 antibody and counterstained with DAPI for nuclei. CD34‐positive and c‐kit‐positive TC were counted in 10 randomly chosen microscopic high‐power fields (hpf; 40× original magnification) per sample. Only the cells with well‐defined nuclei were counted. Counting was performed by two independent observers who were blinded with regard to the sample classification. The final result was the mean of the two different observations for each sample.

### Transmission electron microscopy

For transmission electron microscopy, human corneal specimens were divided into small fragments and fixed in 4% cacodylate‐buffered glutaraldehyde (pH 7.4) at room temperature. Then, the specimens were rinsed in a cacodylate‐buffered solution supplemented with sucrose, post‐fixed in 1% osmium tetroxide (Electron Microscopy Sciences), dehydrated with graded alcohol series, transferred to propylene oxide and embedded in epoxy resin (Epon 812). Semithin sections (2 μm thick) were cut with a RMC MT‐X ultramicrotome (EMME3, Milan, Italy) and stained with a solution of toluidine blue in 0.1 M borate buffer, and then observed under a light microscope. Ultrathin sections (~70 nm thick) of the selected areas were obtained with the same ultramicrotome using a diamond knife and stained with an alcoholic solution of uranyl acetate, followed by an alkaline solution of bismuth subnitrate. Ultrathin sections were examined and photographed under a JEOL JEM‐1010 electron microscope (Jeol, Tokyo, Japan) equipped with a MegaView III high‐resolution digital camera and imaging software (Jeol). Measurements of telopode length and width were performed on at least 20 randomly selected structures per corneal sample. All measurements were carried out on captured transmission electron microscopy photomicrographs employing the ImageJ software (NIH, Bethesda, MD, USA). Results expressed in pixels were converted to their equivalent in μm or nm by the Set Scale function of ImageJ using the scale bar printed on the photomicrograph as reference.

### Statistical analysis

All data are represented as the mean ± S.D. Statistical analysis was performed using the Student's *t*‐test for independent samples. *P* < 0.05 was considered statistically significant. The SPSS software for Windows Version 12.0 (SPSS, Chicago, IL, USA) was used.

## Results

### Normal human cornea

**Figure 1:**
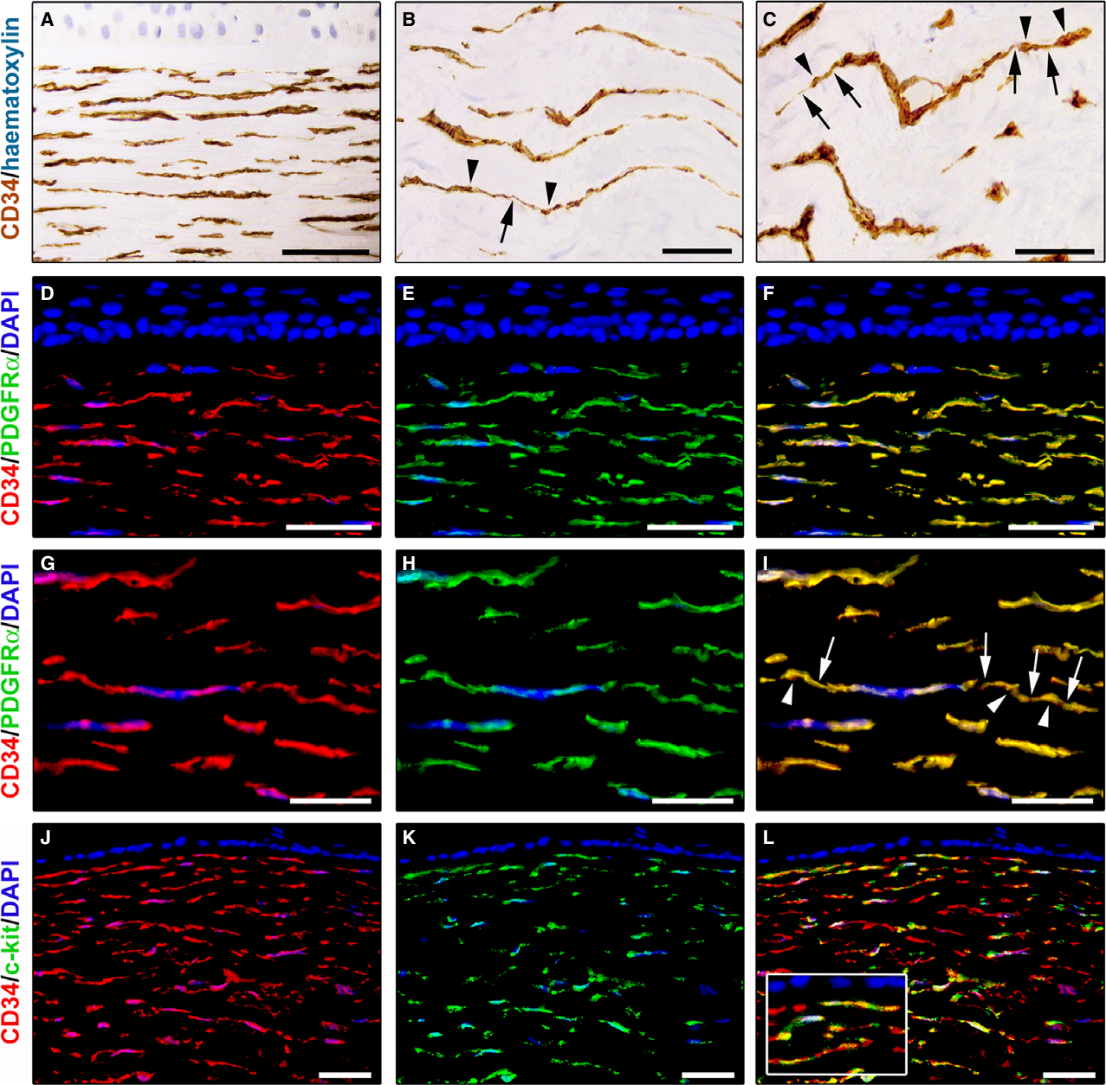
Representative light and fluorescence microscopy photomicrographs of normal human corneal sections. (**A**–**C**) CD34 immunoperoxidase‐based immunohistochemistry with haematoxylin counterstain. (**A**) CD34‐positive stromal cells are orderly arranged and parallel to the corneal surface. (**B** and **C**) At higher magnification, the CD34‐positive stromal cells appear as spindle‐shaped cells with a small oval body and typically two long and thin moniliform cell processes characterized by the alternation of slender segments (*arrows*) and knobs/dilations (*arrowheads*) along their length. (**D**–**I**) Double immunofluorescence labelling for CD34 (red) and PDGFRα (green) with DAPI (blue) counterstain for nuclei. Colocalization of CD34 and PDGFRα in stromal cells gives rise to yellow staining either in the anterior corneal stroma (**D**–**F**) or in the deeper corneal stromal layer (**G**–**I**). CD34^+^/PDGFRα^+^ stromal cells display cell morphologies very evocative for telocytes: a small cell body with very long prolongations (telopodes) characterized by a moniliform silhouette with the alternation of podoms (*arrowheads*) and podomers (*arrows*). (**J**–**L**) Double immunofluorescence labelling for CD34 (red) and c‐kit (green) with DAPI (blue) counterstain for nuclei. Either in the subepithelial corneal stroma or in the deeper stromal layer, numerous CD34‐positive stromal cells coexpress c‐kit. *Inset*: Higher magnification view of CD34^+^/c‐kit^+^ corneal stromal cells. Scale bar: 50 μm (**A**,** D**–**F** and **J**–**L**), 25 μm (**B**,** C** and **G**–**I**).

In normal human corneas, immunoperoxidase‐based immunohistochemistry revealed the presence of numerous CD34‐positive cells throughout the whole thickness of the corneal stroma (Fig. 1A–C). These stromal cells were arranged mainly parallel to the corneal surface and were interspersed between the ECM lamellae (Fig. 1A). In particular, CD34‐positive stromal cells were spindle‐shaped and usually displayed a small oval body and two long and thin varicose prolongations (Fig. 1B and C). Indeed, their cytoplasmic processes typically presented slender segments alternated with small dilated regions (Fig. 1B and C).

We further characterized the immunophenotype of these stromal cells by CD34/PDGFRα double immunofluorescence staining. Double immunolabelling analysis showed colocalization of CD34 and PDGFRα in all corneal stromal cells either in the anterior stroma (Fig. 1D–F) or in the deeper stromal layer (Fig. 1G–I). As displayed in Figure 1D–F, in the anterior stroma, some CD34^+^/PDGFRα^+^ cells were located close to the border of the Bowman's membrane. In the whole corneal stroma, these CD34^+^/PDGFRα^+^ cells appeared orderly arranged parallel to the corneal surface (Fig. 1D–I). At higher magnification, CD34^+^/PDGFRα^+^ corneal stromal cells clearly exhibited long prolongations with a moniliform appearance (Fig. 1G–I).

Double immunostaining for CD34 and c‐kit was also performed on human corneal specimens. As shown in Figure 1J–L, numerous CD34‐positive stromal cells displayed c‐kit immunoreactivity. In particular, CD34^+^/c‐kit^+^ cells were identified either in the subepithelial stroma or in the deeper stroma (Fig. 1J–L).

**Figure 2:**
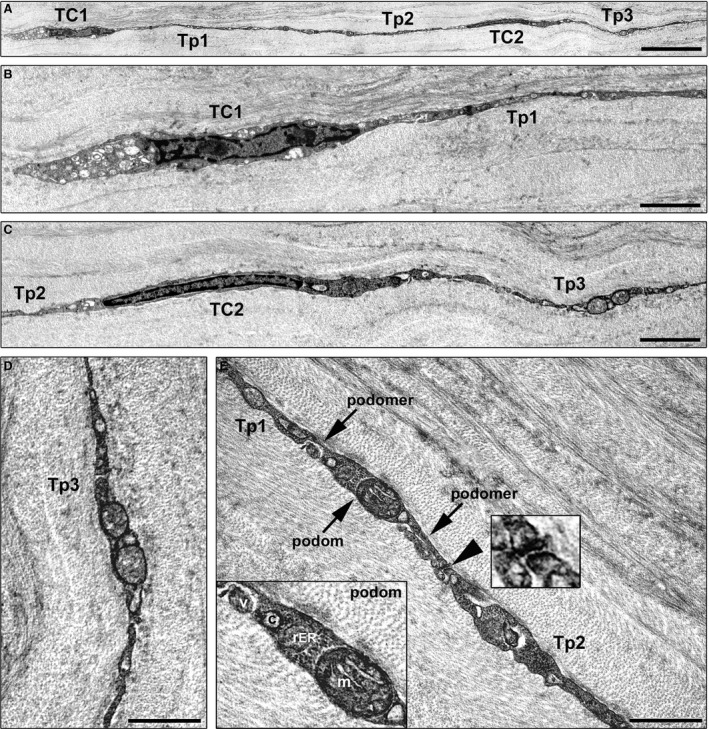
Representative transmission electron microscopy photomicrographs of normal human corneal ultrathin sections. Corneal stromal cells with the typical ultrastructural features of telocytes are observed. (**A**) Two telocytes show a small cell body and very long and thin processes (telopodes) that are collagen‐embedded and connected with intercellular junctions. (**B** and **C**) Corneal telocytes lack a basal lamina and have a scarce cytoplasm surrounding the nucleus with few mitochondria and cisternae of endoplasmic reticulum and a small Golgi apparatus. (**B**–**E**) Telopodes display a moniliform aspect due to the alternation of thin segments (podomers) and dilated cistern‐like portions (podoms) oval or triangular in shape. (**D** and **E**) Podoms contain mitochondria, endoplasmic reticulum cisternae and caveolae. (**E**) Telopodes originating from different telocytes establish gap junctions (*arrowhead*) at the site of contact. *Right inset*: Higher magnification of a gap junction between two telopodes. *Left inset*: Higher magnification of a podom accommodating mitochondria, rough endoplasmic reticulum and caveolae. A shed vesicle is visible near the telopode. TC: telocyte; Tp: telopode; c: caveola; m: mitochondrion; rER: rough endoplasmic reticulum; v: vesicle. Scale bar: 6 μm (**A**), 2 μm (**B** and **C**), 1 μm (**D** and **E**).

Next, we carried out transmission electron microscopy to evaluate in depth the ultrastructural features of these corneal stromal cells. As displayed in Figure 2, corneal stromal cells were embedded in the collagen matrix and appeared as spindle‐shaped bipolar cells characterized by a slender body and usually two very long and thin cytoplasmic prolongations establishing intercellular junctions (Fig. 2A–C). These cells lacked a basal lamina and had a scarce cytoplasm surrounding the nucleus with few mitochondria and cisternae of endoplasmic reticulum and a small Golgi apparatus (Fig. 2B and C). Their collagen‐embedded cellular processes were characteristically very long (up to ~50 μm; 34.6 ± 8.3 μm) and thin, with an uneven calibre and a moniliform aspect due to the alternation of thin segments (89.3 ± 47.2 nm) and dilated portions (274.5 ± 92.4 nm) which contained mitochondria, endoplasmic reticulum cisternae and caveolae (Fig. 2D and E). Moreover, gap junctions, a specialized intercellular connection, were typically observed between the prolongations of corneal stromal cells (Fig. 2E).

Collectively, our findings provide evidence that stromal cells with typical ultrastructural features (*i.e*. cells with telopodes characterized by the alternation of podomers and podoms) and immunophenotypes of TC are present in the human cornea. In addition, our immunohistochemical data support the notion that all human corneal TC are CD34^+^/PDGFRα^+^ and that different TC subtypes may coexist within the corneal stroma, namely c‐kit‐positive and c‐kit‐negative subpopulations.

### Keratoconic human cornea

**Figure 3:**
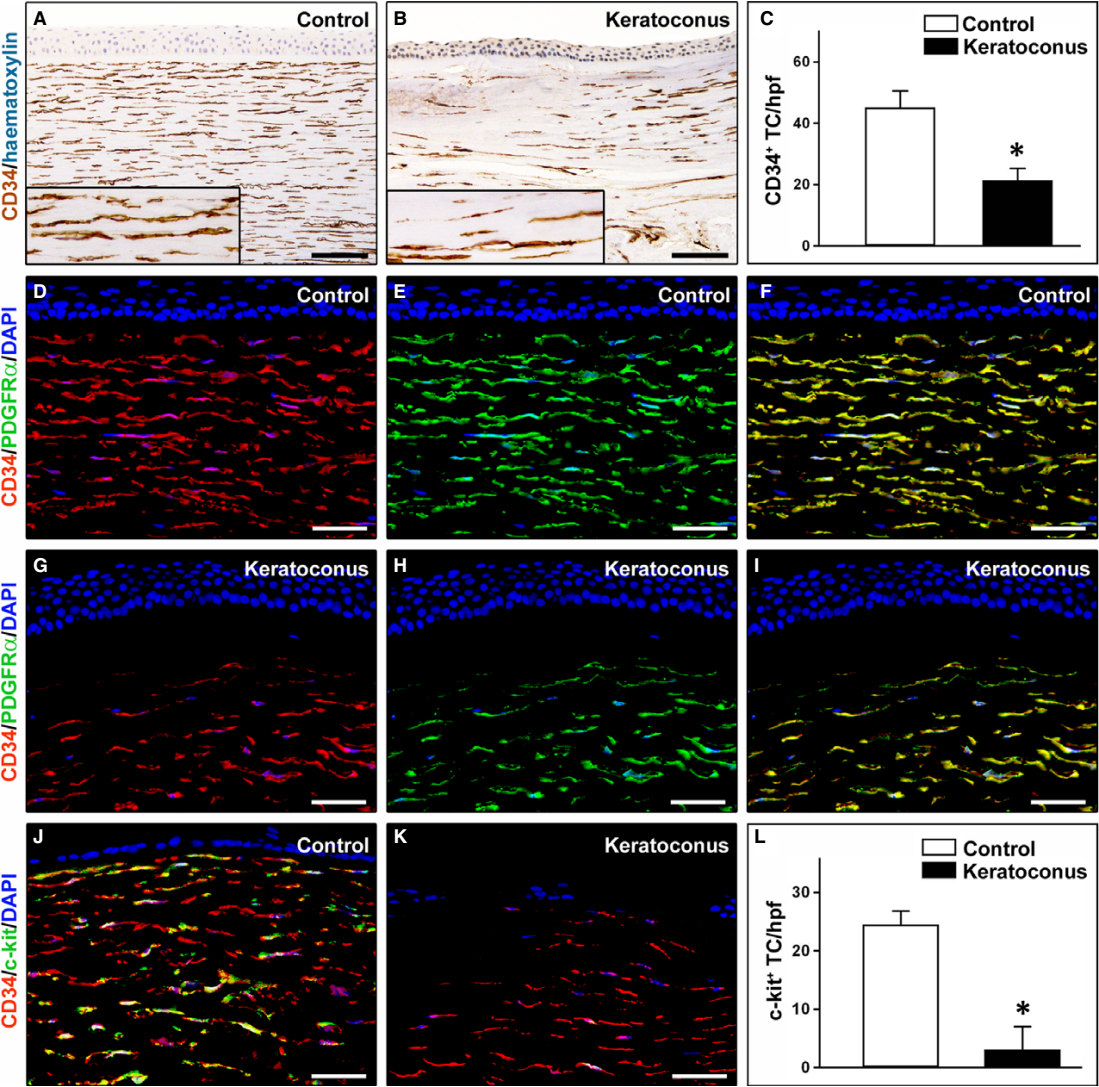
Representative light and fluorescence microscopy photomicrographs of normal and keratoconic human corneal sections. (**A** and **B**) CD34 immunoperoxidase‐based immunohistochemistry with haematoxylin counterstain. (**A**) In control normal corneas, CD34‐positive stromal cells displaying morphological features of telocytes are orderly arranged and parallel to the corneal surface. (**B**) In keratoconus, a patchy loss of CD34‐positive stromal cells is mostly evident in the anterior corneal stroma. *Insets*: Higher magnification views of CD34‐positive corneal stromal cells. (**C**) Results of quantitative analysis of CD34‐positive telocyte counts per high‐power field in the corneal stroma of healthy controls (*n* = 6) and patients with keratoconus (*n* = 6). Data are mean ± S.D. *P < 0.001 *versus* control. (**D**–**I**) Double immunofluorescence labelling for CD34 (red) and PDGFRα (green) with DAPI (blue) counterstain for nuclei. (**D**–**F**) In control normal corneas, CD34^+^/PDGFRα^+^ stromal cells (telocytes) are orderly distributed throughout the stromal compartment. (**G**–**I**) In keratoconic corneas, a patchy loss of CD34^+^/PDGFRα^+^ stromal cells (telocytes) is mainly evident in the subepithelial stroma. (**J** and **K**) Double immunofluorescence labelling for CD34 (red) and c‐kit (green) with DAPI (blue) counterstain for nuclei. (**J**) In control normal corneas, numerous CD34^+^/c‐kit^+^ stromal cells (telocytes) are present throughout the stromal layer. (**K**) In keratoconic corneas, the CD34^+^/c‐kit^+^ stromal cell subpopulation is almost completely lost. (**L**) Results of quantitative analysis of c‐kit‐positive telocyte counts per high‐power field in the corneal stroma of healthy controls (*n* = 6) and patients with keratoconus (*n* = 6). Data are mean ± S.D. *P < 0.001 *versus* control. TC: telocytes; hpf: high‐power field. Scale bar: 100 μm (**A** and **B**), 50 μm (**D**–**K**).

A striking reduction in CD34‐positive stromal cells (hereafter referred to as TC) was found in keratoconic corneas compared with normal corneas (Fig. 3A–C). In particular, in keratoconus TC were unevenly distributed throughout the corneal stroma, with a loss of CD34 immunoreactivity mostly evident in the anterior stroma (Fig. 3B). Indeed, quantitative analysis revealed that the number of CD34‐positive TC/hpf was significantly reduced in keratoconic corneas compared with healthy control corneas (21.4 ± 3.9 *versus* 45.2 ± 5.4, *P* < 0.001; Fig. 3C). These findings were further confirmed by CD34/PDGFRα double immunofluorescence labelling (Fig. 3D–I). Indeed, while in control corneas CD34^+^/PDGFRα^+^ TC were typically found in the whole corneal stroma (Fig. 3D–F), in keratoconus a patchy loss of CD34/PDGFRα immunoreactivity was observed mainly in the subepithelial part of the stroma (Fig. 3G–I). Moreover, double staining for CD34 and c‐kit either confirmed the severe reduction in CD34‐positive TC or revealed an almost complete loss of c‐kit immunoreactivity in keratoconic corneas compared with normal corneas (Fig. 3J–L). In fact, as shown in Figure 3L, the number of c‐kit‐positive TC/hpf was significantly decreased in keratoconus compared with controls (3.1 ± 3.8 *versus* 24.5 ± 2.3, *P* < 0.001).

**Figure 4:**
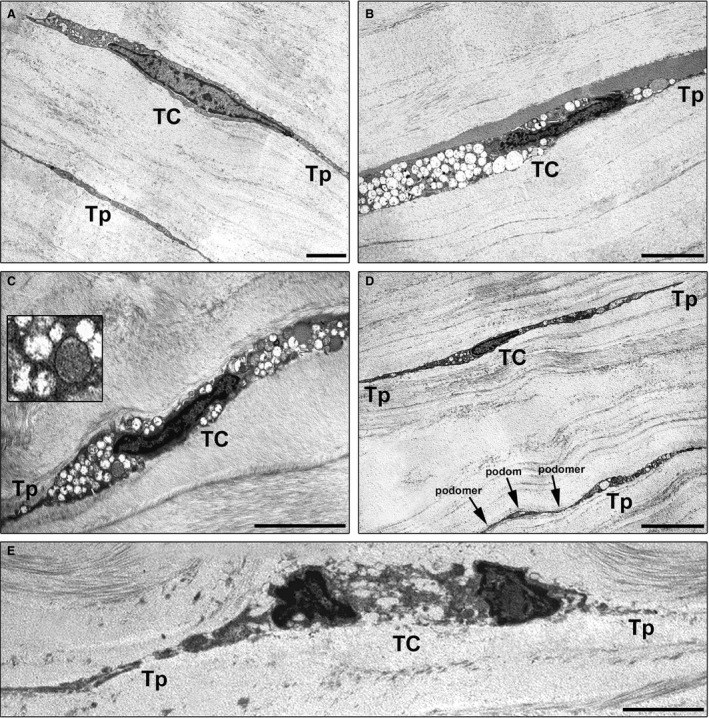
Representative transmission electron microscopy photomicrographs of normal and keratoconic human corneal ultrathin sections. (**A**) In normal human corneas, the typical ultrastructural morphology of telocytes and telopodes is observed. (**B** and **C**) In keratoconic corneas, telocytes display ultrastructural abnormalities including numerous swollen mitochondria, loss of organelles and cytoplasmic vacuolization. *Inset*: Higher magnification of damaged mitochondria. (**D**) In keratoconus, some telocytes and telopodes display a preserved ultrastructural morphology. Note the typical moniliform aspect of telopodes with the alternation of podoms and podomers. (**E**) Shrinkage and shortening of the telopodes along with apoptotic chromatin condensation and nuclear fragmentation are evident in a telocyte from keratoconic corneal stroma. TC: telocyte; Tp: telopode. Scale bar: 2 μm (**A** and **E**), 5 μm (**B**–**D**).

Finally, we performed a comparative ultrastructural analysis between keratoconic and normal corneas under transmission electron microscopy (Fig. 4A–E). At variance with normal corneas, in keratoconus, the majority of TC exhibited ultrastructural abnormalities such as a dark cytoplasm containing numerous swollen mitochondria, loss of organelles and cytoplasmic vacuolization (Fig. 4A–C). As displayed in Figure 4D, some keratoconic TC still showed a preserved ultrastructural morphology. Furthermore, in keratoconus the severity of ultrastructural damages was variable among TC (Fig. 4B, C and E). Indeed, some TC were even characterized by severe degenerative processes including shrinkage and shortening of the telopodes, as well as apoptotic chromatin condensation and nuclear fragmentation (Fig. 4E). Of note, the most severely damaged TC appeared often embedded in a matrix with irregularly distributed collagen bundles (Fig. 4C and E).

## Discussion

In the last years, numerous studies have shown the existence of a novel and peculiar stromal cell type, the TC, in many different organs and tissues of vertebrates including laboratory mammals and humans 10, 11, 12, 13, 14, 15, 16, 17, 18, 19, 20, 21, 22. As far as the ocular system is concerned, a recent report described the presence of TC in the limbus, sclera and uvea of the mouse eye 50. However, to the best of our knowledge, no study has yet described the presence of TC in the human eye and in particular, within the stromal compartment of the cornea.

Here, for the first time, we provide direct evidence for the existence of TC in human cornea by an integrated immunohistochemical and transmission electron microscopy approach. In fact, according to recent literature data 11, 12, 37, 43, 45, 46, corneal TC were mainly recognizable for their typical ultrastructural features (*i.e*. cells with telopodes characterized by the alternation of podomers and podoms) and were immunophenotypically characterized by double immunopositivity for the CD34 and PDGFRα antigens. Noteworthy, the length and width of the telopodes of human corneal TC were consistent with those previously reported for TC in the limbus, sclera and uvea of the mouse eye 50. Moreover, our immunohistochemical findings are in agreement with the view that, although the immunohistochemical profile of TC may vary among different organs and even in the same organ examined 45, 46, at present, CD34/PDGFRα double labelling should be regarded as one of the best marker combination for TC identification under light and fluorescence microscopy 12. Of note, CD34 has been validated as marker of human corneal stromal cells (generally termed keratocytes) in previous studies 6, 7, 8. Thus, taken together, our present findings provide evidence that human corneal stromal cells/keratocytes possess unique ultrastructural features and immunophenotypes which allow the ‘diagnosis’ of TC.

Interestingly, our immunohistochemical results not only indicate that all human corneal TC are CD34^+^/PDGFRα^+^ but also that different TC subtypes coexist within the corneal stroma according to the coexpression of the stemness marker c‐kit/CD117 (*i.e*. c‐kit‐positive and c‐kit‐negative TC subpopulations). Consistent with these findings, previous reports have shown that TC subpopulations sharing the same distinctive ultrastructural features but expressing different immunophenotypical markers may likely play distinct and/or region‐specific roles in various organs, such as in the urinary bladder and in the cardiac striated muscle 45, 46, 47, 48. Moreover, the presence of different subpopulations of corneal stromal cells (*e.g*. keratoblasts, keratocytes and quiescent keratocytes), including cells with stemness features, has previously been suggested in the cornea 2, 51. On the basis of our data, we speculate that in human cornea, the c‐kit‐positive and c‐kit‐negative TC subpopulations might play differential roles. In particular, we propose that the c‐kit‐positive TC might preferentially be involved in corneal regenerative processes presumably acting as precursor cells. This hypothesis is further supported by our immunohistochemical data showing that the c‐kit‐positive TC subpopulation seems to almost completely disappear in human corneas affected by keratoconus, a degenerative corneal ectasia which results in progressive thinning of the cornea and, in more advanced stages, stromal scarring potentially leading to a severe visual impairment and to the necessity of corneal transplantation 6, 7, 9. Therefore, the depletion of c‐kit‐positive TC might contribute to the progression of such a corneal degenerative process by hampering local reparative mechanisms during keratoconus.

It is commonly believed that TC may play an important role in driving organ development and contributing to the maintenance of local tissue homoeostasis and, hence, TC damage and dysfunction may occur in several disorders 23, 24, 25, 26, 27, 28, 29, 30, 31, 32, 33, 52, 53. In this context, we herein demonstrate that the normal morphological features and distribution of TC are severely compromised within the corneal stromal compartment in keratoconic pathology. Indeed, keratoconic corneas were characterized by a severe reduction in TC mainly in the anterior stroma and, at the ultrastructural level, most of the remaining TC displayed degenerative features including numerous swollen mitochondria, loss of organelles, cytoplasmic vacuolization, shrinkage and shortening of the telopodes, as well as apoptotic chromatin condensation and nuclear fragmentation. Of note, these observations are in agreement with previous studies describing an increase in the apoptotic rate within the keratoconic corneal stroma as detected by TUNEL assay 6, 7. According to the possible functions which have been proposed for TC in a variety of organs 11, 12, 46, the regular distribution of TC and their telopodes that we observed throughout the whole normal corneal stroma might contribute to the correct assembly and maintenance of a highly organized collagenous matrix which is essential for conferring the adequate corneal transparency and mechanical stability. In addition, we found that in the normal cornea, TC establish homocellular contacts formed by gap junctions, highly specialized membrane structures which allow the exchange of small molecular mediators involved in intercellular signalling and thus contributing to the maintenance of tissue homoeostasis 38, 39. As suggested in other pathologies 23, 25, 26, 27, 28, 29, the reduction and loss of TC might therefore contribute to the altered organization of the ECM ultimately resulting into corneal dysfunction in keratoconus.

In conclusion, our data provide the first demonstration that TC are present in the human cornea and also highlight their possible implication in corneal pathology. Further *in vitro* and *in vivo* studies are warranted to clarify the biological functions of TC in corneal repair and regeneration. In particular, deciphering the differential roles of corneal TC subtypes might provide new insights into their possible therapeutic utility in the context of corneal regenerative medicine.

## Conflict of interests

The authors confirm that there are no conflict of interests.

## Author contribution

Marini and Manetti designed the study; Marini, Mencucci, Rosa, Favuzza, Guasti, Ibba‐Manneschi and Manetti performed acquisition of data; Marini, Ibba‐Manneschi and Manetti performed interpretation of data; and Marini, Ibba‐Manneschi and Manetti performed manuscript preparation.
